# The clinical value of ultrasound radiomics combined with clinical data nomogram in predicting cervical lymph node metastasis of papillary thyroid carcinoma

**DOI:** 10.3389/fonc.2026.1811434

**Published:** 2026-10-07

**Authors:** Xuxia Zhang, Biyun Sun, Feng Jiang, Yuxin Chu, Xingyu Wang, Wenhui Sun, Tianjun Wei, Ruiyang Hong, Qianqing Ma

**Affiliations:** 1 Department of Ultrasound, Yijishan Hospital, The First Affiliated Hospital of Wannan Medical University, Wuhu, China; 2 The First Affiliated Hospital of Soochow University, Suzhou, China

**Keywords:** cervical lymph node metastasis, nomogram, Radscore, risk prediction, ultrasound radiomics

## Abstract

**Objectives:**

To evaluate the predictive value of a nomogram incorporating ultrasound and clinical features for cervical lymph node metastasis (CLNM) in patients with thyroid cancer.

**Methods:**

A retrospective analysis was conducted on patients pathologically diagnosed with papillary thyroid carcinoma (PTC) between April 2024 and January 2025. Radiomics features were extracted from two-dimensional thyroid ultrasound images, and feature selection was performed using the least absolute shrinkage and selection operator (LASSO). Five classifiers were compared, and logistic regression was selected to construct the radiomics model. A clinical-radiomics model integrating the radiomics score and clinical predictors, together with a standalone clinical model, was developed using multivariable logistic regression. Model discrimination, calibration, and clinical utility were evaluated in a held-out internal validation set.

**Results:**

Sex, Radscore, age, and thyroid stimulating hormone (TSH) levels were identified as independent predictors of CLNM in patients with PTC. Based on these variables, radiomics, clinical, and clinical radiomics models were constructed; the models achieved area under the curve (AUC) values of 0.735, 0.792, and 0.867, respectively, in the validation set. DeLong’s test and decision curve analysis (DCA) demonstrated that the clinical radiomics model outperformed the radiomics model in the validation set, offering a higher net clinical benefit.

**Conclusion:**

A nomogram integrating ultrasound imaging and clinical data effectively predicts the risk of CLNM in patients with PTC and may assist clinicians in developing individualized treatment strategies.

## 1 Introduction

The global incidence of thyroid cancer has increased in recent years (1), with papillary thyroid carcinoma (PTC) representing the predominant histological subtype, accounting for approximately 89.1% of cases (2). It is now one of the most prevalent malignant tumors of the endocrine system (3). While PTC typically exhibits low malignant potential, with a 5-year survival rate over 95%, central lymph node metastasis significantly influences recurrence, distant metastasis risk, and overall survival in thyroid cancer. Lateral lymph node metastasis is detected in approximately 20%–50% of patients with PTC at the time of diagnosis, and 7.4%–9.7% of cases are reportedly missed. In addition, cervical metastatic lymph nodes (CLNM) occurs in up to 68% of cases (4, 5). The recurrence risk among patients with metastasis can reach around 60% (6), thereby significantly impacting surgical planning and long-term quality of life (7, 8). Therefore, accurate preoperative evaluation of the pathological status of cervical lymph nodes (CLNs) is essential to prevent effects of overtreatment (e.g., complications such as bleeding, recurrent laryngeal nerve injury, and hypoparathyroidism caused by unnecessary lymph node dissection) or undertreatment (such as postoperative recurrence due to missed diagnosis of metastatic lesions) (9, 10). This assessment is also a core link in the implementation of individualized diagnostic and treatment strategies for patients with PTC (11).

Currently, clinical assessment of CLNM in PTC remains challenging (12). Conventional ultrasound (US), while being the preferred imaging method, has diagnostic accuracy heavily reliant on the operator’s experience and subjective interpretation (13–15). Its performance is further constrained by the anatomical characteristics of lymph nodes, such as their deep location and scattered distribution, resulting in a detection sensitivity of only 28% for central compartment metastasis and an overall diagnostic accuracy of approximately 63% (16). Fine-needle aspiration cytology (FNAC) serves as a valuable supplement to pathological diagnosis but has a misdiagnosis rate of 20%-30% and limited ability to detect small metastatic lesions. Clinical data, including age, tumor size, and thyroid function indicators, provide baseline risk information (14, 17–19); however, the predictive efficacy of individual factors is inadequate for precise assessment (5, 13). Taken together, the limitations of these existing modalities highlight a clear clinical requirement for a noninvasive, efficient, and robust comprehensive prediction tool.

The emergence of radiomics technology offers a novel approach to overcoming these challenges. By applying computer-based algorithms, radiomics enables the extraction of quantitative features from medical images that are not visible to the naked eye, thereby transforming image information into objective data and significantly enhancing information mining depth (20, 21). As a visualized clinical prediction tool, a nomogram can integrate multi-dimensional predictive factors and intuitively quantify an individual’s risk of metastasis through cumulative score calculation (8, 22, 23). Based on this, this study aims to construct a nomogram-based prediction model that combines ultrasound radiomics with clinical data. By integrating quantitative ultrasound features with clinical data such as patients’ basic information, immunohistochemical factors, and pathological indicators, this study systematically evaluates the clinical value of the proposed model for predicting CLNM in PTC. This paper aims to provide a new method for accurate preoperative assessment, support optimized surgical decision-making, and promote the development of PTC diagnosis and treatment towards precision and individualized care. The manuscript was prepared according to the TRIPOD checklist.

## 2 Materials and methods

### 2.1 Data source and study population

This retrospective study was approved by the Institutional Review Board of the First Affiliated Hospital of Wannan Medical University (approval No. 2026-25), with the requirement for informed consent waived. Clinical data and ultrasound images obtained during routine care between April 2024 and January 2025 were retrospectively reviewed. No additional interventions were performed.

A retrospective analysis was performed on 158 patients with pathologically confirmed PTC treated at our hospital between April 2024 and January 2025. These patients contributed 170 target lesions. Inclusion criteria were: (1) PTC confirmed by surgery and pathology; for unilateral multifocal disease, the largest lesion in the affected lobe was selected, whereas for bilateral disease, the largest lesion in each lobe was selected; (2) cervical lymph node status verified by lymph node dissection; and (3) ultrasound examination and BRAF testing performed within two weeks before surgery. Exclusion criteria were: (1) preoperative radiotherapy or chemotherapy; and (2) incomplete clinical information or inadequate ultrasound image quality. **Figure 1** shows patient and lesion selection.

**Figure 1:**
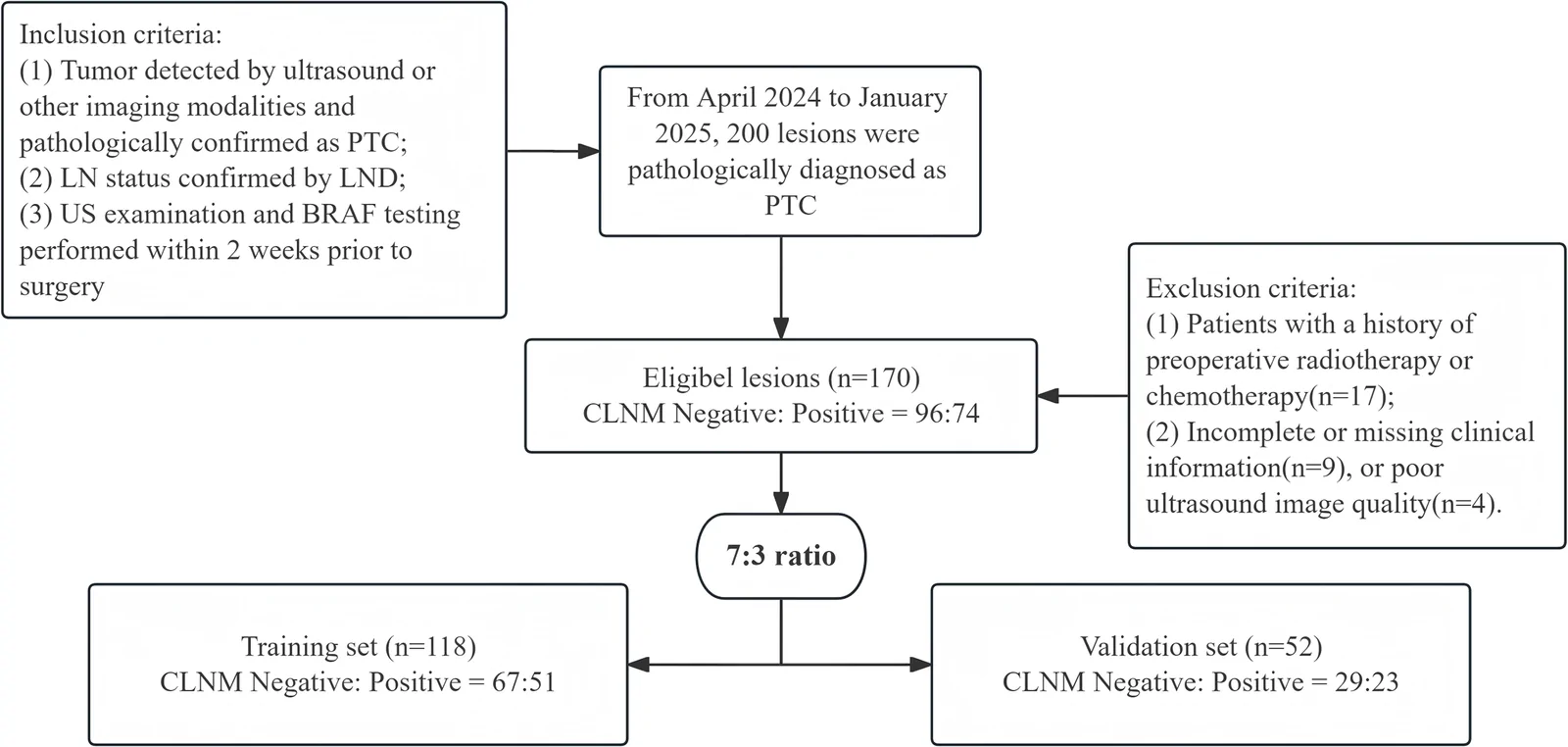
The procedure of recruitment and partition of datasets.

Electronic medical records were used to obtain age, sex, nodule pathology, lymph node status, BRAF status, Bethesda category, thyroid-stimulating hormone (TSH), thyroglobulin antibody, and thyroid peroxidase antibody. The target lesion was used as the unit of analysis. For unilateral multifocal disease, the largest lesion in the affected lobe was selected; for bilateral disease, the largest lesion in each lobe was included, so some patients contributed two lesions. Patients were assigned to either the training or internal validation set, with all lesions from the same patient kept in the same set. The models were subsequently developed and evaluated using the 170 included lesions.

### 2.2 Collection of ultrasonic data

Ultrasound images of thyroid tumors were acquired using various systems: the Resona 7 and Resona DC-8 from Mindray Medical International (Shenzhen, China) equipped with a linear array probe (L14–5 W) at 4.0–14.0 MHz, the Philips EPIQ5 and Affiniti 70W from Philips Healthcare (Eindhoven, The Netherlands) with a linear array probe (eL18–4 W) at 4.0–18.0 MHz, and the MyLab Class C from Esaote Medical International (Genoa, Italy) with a linear array probe (LA523 W) at 4.0–13.0 MHz. These examinations were conducted by physicians with 10 to 12 years of experience in thyroid ultrasound imaging.

During ultrasound examination, tumor characteristics were assessed and classified according to the ACR Thyroid Imaging Reporting and Data System (TIRADS) while capturing images. The evaluated ultrasound features included tumor size, location, composition, echogenicity, margins, shape, orientation, and microcalcifications.

### 2.3 ROI delineation, image-based feature extraction, and radiomics model development

Regions of interest (ROIs) were manually delineated on ultrasound images using ITK-SNAP 3.8.0. A random subset of 70 lesions was selected for reproducibility assessment. A radiologist with 12 years of thyroid ultrasonography experience performed the initial delineation and repeated the delineation two weeks later for assessment of intraobserver reproducibility. A second radiologist with 10 years of experience independently delineated the same lesions for assessment of interobserver reproducibility. All radiologists were blinded to pathology and clinical information. Intraobserver and interobserver intraclass correlation coefficients (ICCs) were evaluated separately, and features with ICC values greater than 0.80 in both assessments were retained for subsequent analysis.

Radiomics features were extracted from single two-dimensional ultrasound frames using PyRadiomics in Python 3.7. The images were stored as singleton-slice NIfTI files, with the third dimension equal to one, and no volumetric ultrasound data were analyzed. Candidate features were calculated from the original images and from wavelet, Laplacian-of-Gaussian, square, square-root, logarithm, exponential, gradient, and LBP2D transforms. The automated extraction also generated variables with the LBP3D prefix because the singleton-slice files were read as three-dimensional SimpleITK objects. These variables did not represent true volumetric texture, and none was retained in the final logistic radiomics model. A total of 1,688 candidate features were extracted from each image.

Before model construction, all extracted features were normalized. Redundant features were removed using Spearman correlation, retaining one feature from each pair with an absolute correlation coefficient greater than 0.90. The LASSO tuning parameter was selected by tenfold cross-validation within the training set, and features with nonzero coefficients were retained. Logistic regression (LR), random forest (RF), support vector machine (SVM), XGBoost, and k-nearest neighbors (KNN) were compared on the held-out validation set.

We thoroughly assessed the predictive performance of models constructed using these five machine learning classifiers using the area under the receiver operating characteristic (ROC) curve (AUC), along with accuracy, sensitivity, and specificity metrics. The classifier with the best overall performance was selected as radiomics model, and the corresponding radscore was computed for each patient.

### 2.4 Construction of the clinical model and clinical radiomics model

Univariable logistic regression was used to screen sex, age, BRAF status, Bethesda category, TSH, TgAb, TPOA, lesion count, location, lesion diameter, margin, capsular proximity, echogenicity, orientation, microcalcifications, and Radscore. Variables with (P<0.05) were entered into multivariable logistic regression, from which the independent predictors were identified. TSH was included as a continuous variable.

### 2.5 Model evaluation and nomogram generation

The diagnostic performance of radiomics model, clinical model and clinical-radiomics model was assessed using the AUC, along with measures of accuracy, sensitivity, and specificity. Decision curve analysis (DCA) plots were performed to visualize the net benefit of clinical decision-making. Calibration was assessed using calibration curves in the training and validation sets. The Hosmer–Lemeshow goodness-of-fit test was additionally performed in the training set. A nomogram based on clinical-radiomics model was created to improve clinical applicability.

### 2.6 Statistical analysis

Statistical analyses were conducted using R version 4.1.2 and SPSS version 25.0. Clinical characteristics were assessed for normality and homogeneity of variance. Normally distributed continuous variables are presented as mean ± standard deviation (SD), whereas non-normally distributed variables are reported as median (interquartile range). Chi-squared or Fisher’s exact tests were used to compare categorical variables between the training and validation sets. T-tests or Mann–Whitney U tests were employed to compare continuous variables. Statistical significance was determined using two-tailed tests, with P < 0.05 indicating significance. For the exploratory TSH threshold analysis, the ROC curve and Youden index were calculated in the training set only.

## 3 Results

### 3.1 Baseline characteristics

This study included 158 patients who contributed 170 target thyroid lesions. The training set comprised 111 patients and 118 target lesions, whereas the internal validation set comprised 47 patients and 52 target lesions. No patient was represented in both sets. **Table 1** presents the baseline characteristics of all patients.

**Table 1:** Baseline characteristics in the training and internal validation cohorts.

Variable		Training set	Validation set	P value
CLNM	Negative	67 (56.8%)	29 (55.8%);	0.9026
	Positive	51 (43.2%)	23 (44.2%)	
Sex	Female	87 (73.7%)	45 (86.5%)	0.0647
	Male	31 (26.3%)	7 (13.5%)	
Radscore		0.43 (0.25, 0.58)	0.39 (0.21, 0.63)	0.8671^*^
Age		47.5 (35.25, 52.75)	50.5 (40.75, 54.25)	0.0929^*^
BRAF	Negative	12 (10.2%)	2 (3.8%)	0.2313^#^
	Positive	106 (89.8%)	50 (96.2%)	
Bethesda	3	7 (5.9%)	4 (7.7%)	0.8154^#^
	4	2 (1.7%)	1 (1.9%)	
	5	103 (87.3%)	46 (88.5%)	
	6	6 (5.1%)	1 (1.9%)	
Lesion Count	Single	81 (68.6%)	32 (61.5%)	0.3659
	Multiple	37 (31.4%)	20 (38.5%)	
Location	Right Lobe	63 (53.4%)	20 (38.5%)	0.1499
	Left Lobe	49 (41.5%)	30 (57.7%)	
	Isthmus	6 (5.1%)	2 (3.8%)	
Maximum Tumor Diameter		8 (6.03, 10)	8.5 (6.5, 11)	0.1887^*^
Tumor Size	≤5mm	14 (11.9%)	3 (5.8%)	0.2222
	>5mm	104 (88.1%)	49 (94.2%)	
Margin	Smooth	62 (52.5%)	33 (63.5%)	0.1864
	Irregular	56 (47.5%)	19 (36.5%)	
Echogenicity	Marked Hypoechoic	10 (8.5%)	5 (9.6%)	0.6562^#^
	Hypoechoic	95 (80.5%);	40 (76.9%)	
	Isoecho	8 (6.8%)	3 (5.8%)	
	Hyperechoic	0(0%)	1 (1.9%)	
	Mixed Echo	0(0%)	3 (5.8%)	
Orientation	Parallel	31 (26.3%)	12 (23.1%)	0.6589
	Nonparallel	87 (73.7%)	40 (76.9%)	
Microcalcification	Absent	49 (41.5%)	21 (40.4%)	0.8892
	Presence	69 (58.5%)	31 (59.6%)	
TSH		2.36 (1.55, 3.16)	1.75 (1.19, 2.67)	0.0585^*^
Tgab	Negative	93 (78.8%)	40 (76.9%)	0.7831
	Positive	25 (21.2%)	12 (23.1%)	
TPOA	Negative	87 (73.7%)	40 (76.9%)	0.6589
	Positive	31 (26.3%)	12 (23.1%)	

*Mann-Whitney U test, ^#^Fisher’s Exact Test, CLNM cervical lymph node metastasis.

### 3.2 Screening of radiomics features and development of a radiomics model

A total of 1,688 features were extracted from ultrasound images. After excluding features with intra-observer and inter-observer ICCs < 0.8, 1,270 features remained for further analysis. Following Spearman correlation testing, 122 features were retained. These feature parameters underwent LASSO screening, ultimately preserving eight features (**Figure 2**).

**Figure 2:**
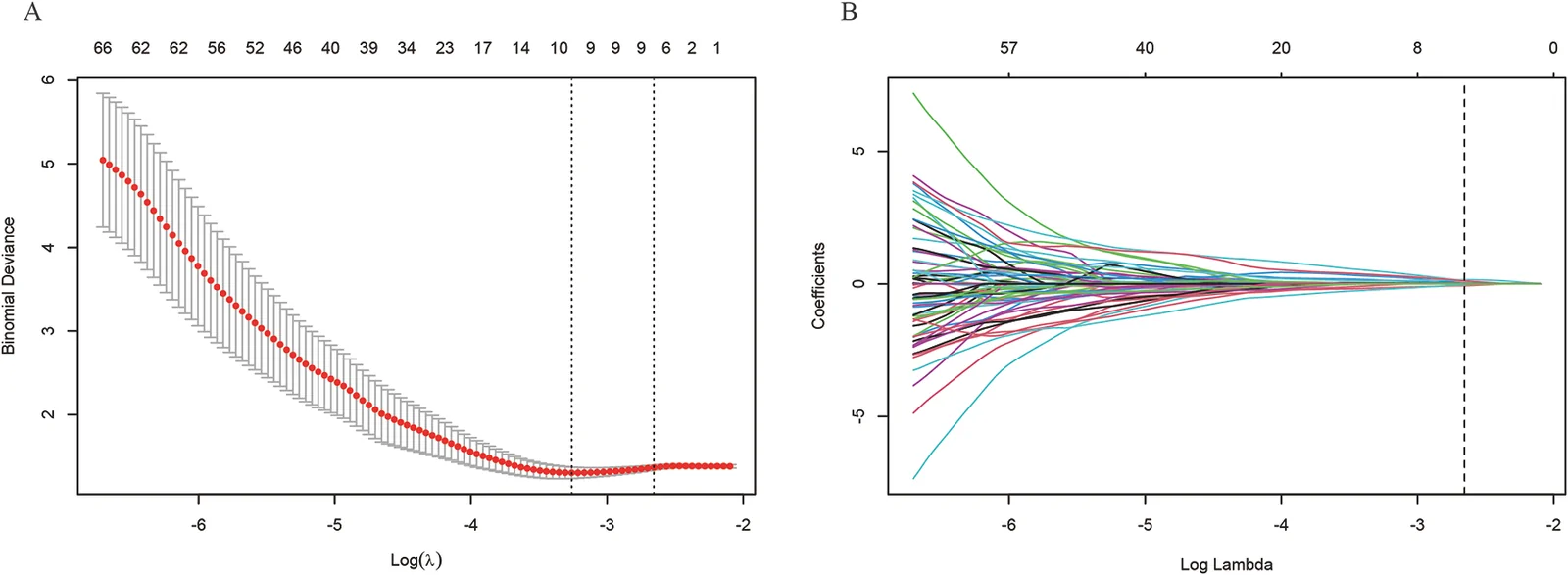
Radiomics feature selection using the least absolute shrinkage and selection operator (LASSO) and logistic regression. **(A)** Tuning parameter (λ) selection in the LASSO model using tenfold cross-validation via minimum criteria. **(B)** LASSO coefficient profiles of the radiomics features.

Radiomics model was constructed using these selected features with five classifiers: LR, RF, SVM, XGBoost, and KNN. The performance of the five models is summarized in **Table 2**. In the training set, the RF and XGBoost models had the highest diagnostic AUC, reaching 1.00. However, in the validation set, the AUC of the two models decreased to 0.655 and 0.584, respectively, indicating overfitting during training and limited generalizability. In contrast, the LR model demonstrated consistent and stable performance across both the training and validation sets. Consequently, based on the diagnostic performance across the two datasets, the LR model was selected as radiomics model for this study, and four features were retained in this model Using this model, we computed the radiomics score for each patient.

**Table 2:** Performance evaluation of five classifiers.

	AUC	Accuracy	Sensitivity	Specificity
Train				
Logistic Regression	0.769 (0.684-0.854)	0.712	0.706	0.716
Random Forest	1.000 (1.000-1.000)	1.000	1.000	1.000
SVM	0.7795 (0.683-0.863)	0.763	0.549	0.925
XGBoost	1.000 (1.000-1.000)	1.000	1.000	1.000
KNN	0.851 (0.775-0.909)	0.771	0.647	0.866
Validation				
Logistic Regression	0.735 (0.595-0.874)	0.712	0.783	0.655
Random Forest	0.655 (0.500-0.796)	0.558	0.4348	0.6552
SVM	0.667 (0.518-0.808)	0.673	0.478	0.828
XGBoost	0.584 (0.425-0.732)	0.577	0.435	0.690
KNN	0.603 (0.452-0.743)	0.558	0.435	0.655

SVM, Support Vector Machine; KNN, k-Nearest Neighbor; AUC, area under the curve.

The formula for calculating each patient’s Radscore according to this model is as follows:


Radscore=−0.3668+1.1344∗square_glszm_SmallAreaLowGrayLevelEmphasis+1.1492∗exponential_glszm_GrayLevelNonUniformity−0.9444∗logarithm_ngtdm_Contrast+0.8942∗squareroot_glcm_JointEntropy

### 3.3 Clinical model and clinical-radiomics model construction

The ULR results for sex, age, BRAF status, Bethesda category, TSH, TgAb, TPOA, lesion count, location, lesion diameter, margin, capsular proximity, echogenicity, orientation, microcalcifications, and Radscore are presented in **Table 3** as exploratory screening analyses. MLR identified sex, age, TSH, and Radscore as independent predictors of CLNM. Sex, age, and TSH were used in the clinical model, and these variables plus Radscore were used in the clinical-radiomics model. In the training set, the Hosmer–Lemeshow test yielded χ^2^ = 11.929 (P = 0.154) for the clinical model and χ^2^ = 14.660 (P = 0.066) for the clinical-radiomics model.

**Table 3:** univariate and multivariate logistic regression analysis of CLNM status in training set.

Variable		Univariate logistic regression		Multivariate logistic regression			
				Clinical model		Clinical-radiomics model	
		OR (95% CI)	P	OR (95% CI)	P	OR (95% CI)	P
Sex	Male (Female ref.)	3.99 (1.702-9.89)	0.002*	3.493 (1.33-9.745)	0.013*	4.078 (1.293-14.143)	0.020*
Radscore		157.76 (21.914-1587.714)	0.000*	NA	NA	298.751 (26.935-5352.354)	0.000*
Age		0.917 (0.877-0.954)	0.000*	0.921 (0.88-0.96)	0.000*	0.905 (0.855-0.951)	0.000*
BRAF	Positive (Negative ref.)	2.482 (0.696-11.658)	0.191	NA	NA	NA	NA
Bethesda	3 (ref.)		0.603	NA	NA	NA	NA
	4	0.000	0.999	NA	NA	NA	NA
	5	0.538 (0.114-2.526)	0.432	NA	NA	NA	NA
	6	1.5 (0.156-14.42)	0.725	NA	NA	NA	NA
Lesion Count	Multiple (Single ref.)	1.175 (0.534-2.573)	0.686	NA	NA	NA	NA
Location	Right Lobe (ref.)		0.876	NA	NA	NA	NA
	Left Lobe	1.16 (0.546-2.465)	0.700	NA	NA	NA	NA
	Isthmus	1.423 (0.266-7.613)	0.680	NA	NA	NA	NA
Maximum Tumor Diameter		1.08 (0.999-1.178)	0.063	NA	NA	NA	NA
Tumor Size	>5mm (≤5mm ref.)	5.345 (1.369-35.448)	0.034*	NA	NA	NA	NA
Margin	Irregular (Smooth ref.)	1.282 (0.618-2.674)	0.504	NA	NA	NA	NA
Echogenicity	Marked Hypoechoic (ref.)		0.202	NA	NA	NA	NA
	Hypoechoic	0.312 (0.076-1.28)	0.106	NA	NA	NA	NA
	Isoecho	0.000	0.999	NA	NA	NA	NA
	Hyperechoic	1.714 (0.131-22.513)	0.682	NA	NA	NA	NA
	Mixed Echo	0.312 (0.076-1.28)	0.202	NA	NA	NA	NA
Orientation	Non-Parallel (Parallel ref.)	0.752 (0.329-1.725)	0.500	NA	NA	NA	NA
Microcalcification	Present (Absent ref.)	1.365 (0.651-2.9)	0.412	NA	NA	NA	NA
TSH		1.715 (1.276-2.426)	0.001*	1.596 (1.166-2.323)	0.008	1.464 (1.03-2.202)	0.048*
TgAb	Positive (Negative ref.)	0.683 (0.264-1.675)	0.413	NA	NA	NA	NA
TPOA	Positive (Negative ref.)	0.777 (0.329-1.78)	0.555	NA	NA	NA	NA

CI, confidence interval; NA, not applicable; OR, odds ratio; Ref, reference value; *P<0.05.

In the training set, TSH alone yielded an AUC of 0.680 (95% CI 0.584-0.777). The Youden-optimal cutoff was 1.807 mU/L. At this cutoff, accuracy was 0.627, sensitivity was 0.824, specificity was 0.478, positive predictive value was 0.545, and negative predictive value was 0.780.

### 3.4 Model evaluation and nomogram plotting

**Table 4** summarizes the performance of the radiomics, clinical, and clinical-radiomics models. In both the training and validation sets, the clinical-radiomics model had the highest AUC. In the training set, DeLong tests indicated higher AUCs for the clinical-radiomics model than for the radiomics model (Z = 3.248, P = 0.001) and the clinical model (Z = 2.297, P = 0.022). In the validation set, the clinical-radiomics model had a higher AUC than the radiomics model (0.867 vs 0.735; Z = 2.467, P = 0.014). **Figures 3A, B** show the ROC curves, and **Figures 3C, D** show the corresponding decision-curve analyses for the training and validation sets.

**Table 4:** Performance of three models in the training and validation sets.

Group	Model	AUC (95% CI)	Accuracy	Sensitivity	Specificity
Training set	Radiomics Model	0.769 (0.684-0.854)	0.712	0.706	0.716
	Clinical Model	0.81 (0.729-0.891)	0.763	0.745	0.776
	Clinical Radiomics Model	0.886 (0.824-0.948)	0.831	0.863	0.806
Validation set	Radiomics Model	0.735 (0.595-0.874)	0.712	0.783	0.655
	Clinical Model	0.792 (0.67-0.913)	0.712	0.826	0.621
	Clinical Radiomics Model	0.867 (0.764-0.969)	0.827	0.826	0.828

AUC, area under the curve; CI, confidence interval.

**Figure 3:**
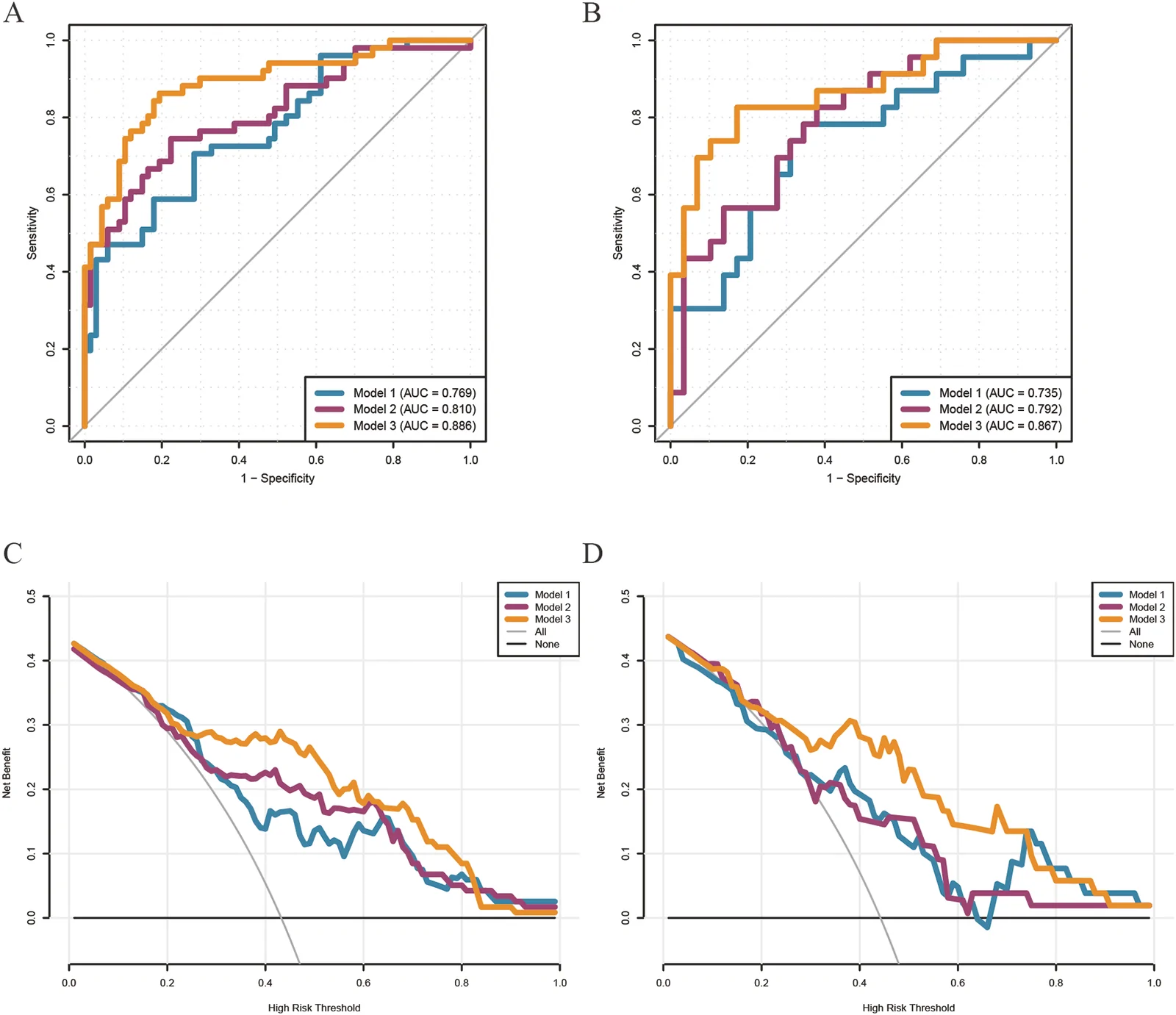
ROC and decision curves for the radiomics, clinical, and clinical-radiomics models. **(A)** ROC curves in the training set; **(B)** ROC curves in the validation set; **(C)** decision curves in the training set; and **(D)** decision curves in the validation set.

Clinical and clinical-radiomics nomograms were constructed based on clinical model and clinical-radiomics model (**Figures 4A, B**). The training-set calibration curves were relatively close to the ideal diagonal, whereas the validation curves showed deviations in the intermediate predicted-probability range (**Figures 4C, D**). A representative application of the clinical-radiomics nomogram is provided in **Supplementary Figure S1**.

**Figure 4:**
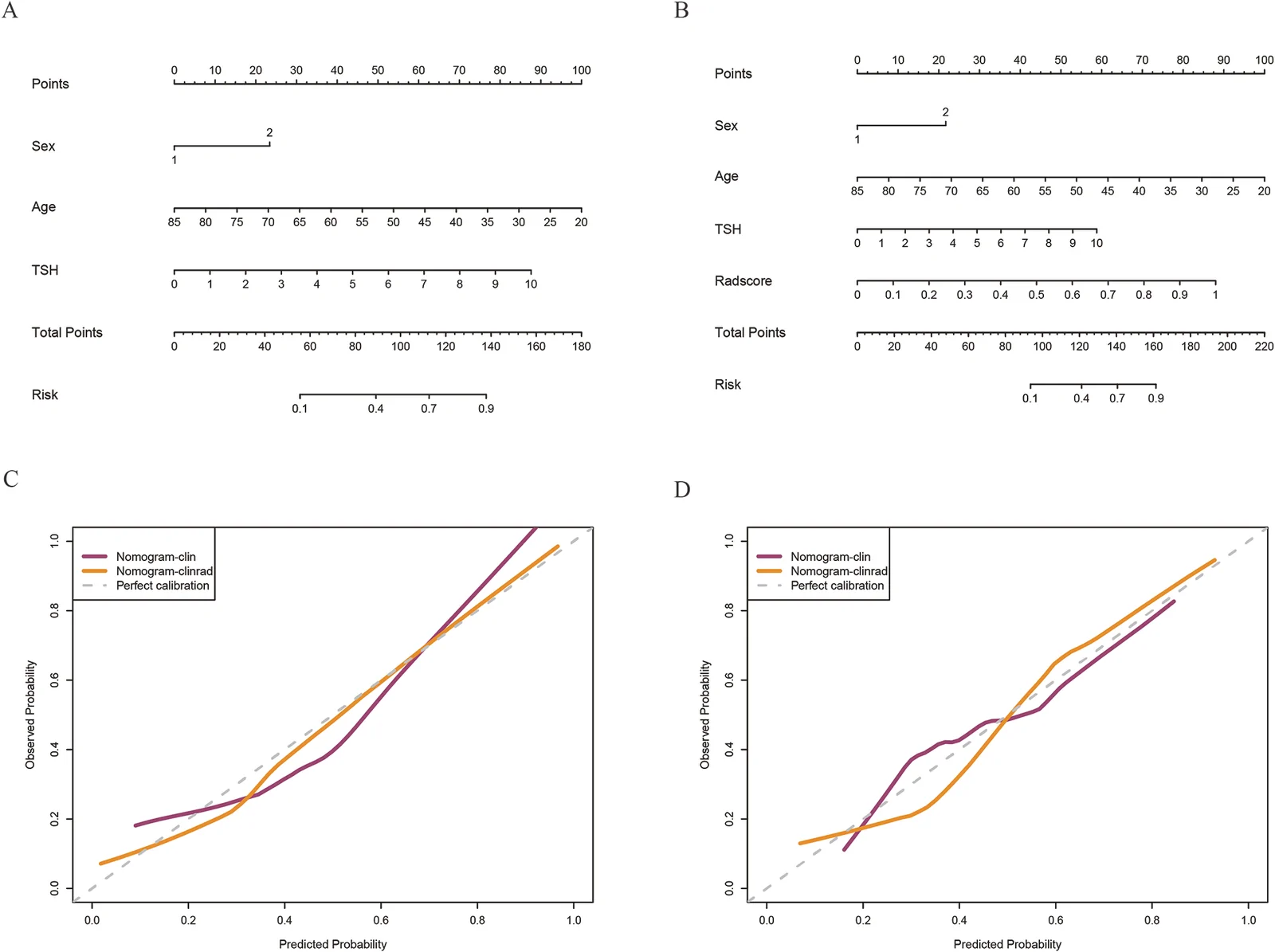
Clinical and clinical-radiomics nomograms and calibration curves. **(A)** Clinical nomogram; **(B)** clinical-radiomics nomogram; **(C)** calibration curves in the training set; and **(D)** calibration curves in the validation set. Model 1, model 2, Model 3 are radiomics, clinical, and clinical-radiomics model, respectively.

## 4 Discussion

This study developed a clinical-radiomics model integrating sex, age, TSH, and Radscore to predict CLNM in PTC. The model achieved an AUC, accuracy, sensitivity, and specificity of 0.886, 83.1%, 86.3%, and 80.6%, respectively, in the training set and 0.867, 82.7%, 82.6%, and 82.8% in the internal validation set. The clinical-radiomics model outperformed the radiomics and clinical models in the training set and the radiomics model in the validation set. Decision-curve analysis showed that it provided the highest net benefit across most threshold probabilities. However, the validation calibration curves deviated from the ideal diagonal at intermediate predicted probabilities, suggesting that the estimated risks should be interpreted cautiously and further evaluated in an external cohort.

Tumor diameter is a key clinical indicator for evaluating CLNM. In clinical practice, thyroid tumor diameter is generally considered to be positively correlated with the degree of malignancy (5, 24). Numerous studies, including those by Fu et al. (3) have validated the predictive significance of tumor size for CLNM. Wang et al. demonstrated that patients with PTC and tumor diameters ≥ 1.0 cm had a threefold higher risk of CLNM than did those with tumor diameters < 1.0 cm (14). Additionally, another study demonstrated a significant correlation between increased tumor volume and a higher number of CLNM. In this study, tumor diameter was excluded from the final variable system for both clinical model and clinical-radiomics model. Despite univariate analysis indicating a higher probability of CLNM in lesions larger than 5 mm, multivariate logistic regression revealed significant information overlap between tumor diameter and other variables, leading to its exclusion by the algorithm.

Among the clinical predictors, sex was associated with CLNM. With female patients as the reference group, male patients had higher odds of metastasis in the clinical-radiomics model (OR = 4.078, 95% CI 1.293-14.143; P = 0.020). This direction is consistent with the higher male risk reported by Xue et al. (17) The study found a significantly higher proportion of males in the Papillary thyroid microcarcinoma (PTMC, diameter ≤ 1.0cm) CLNM group compared to those in the non-metastasis group. This discrepancy may be attributable to regional variations in genetic background and clinical features (24). Further validation in larger, multicenter prospective studies is required to confirm the reliability of this association.

Younger age was associated with a higher risk of CLNM. In the clinical-radiomics model, the OR for age was 0.905 per additional year (95% CI 0.855-0.951; P < 0.001), corresponding to an approximately 8.3% reduction in the odds of CLNM for each one-year increase in age. Previous studies have also linked younger age with higher nodal metastatic risk (25). TSH may promote PTC cell proliferation and invasion through signaling pathways including PI3K-AKT (26). In the present study, the training-set Youden-optimal TSH threshold was 1.807 mU/L, with an AUC of 0.680, accuracy of 0.627, sensitivity of 0.824, specificity of 0.478, PPV of 0.545, and NPV of 0.780. Similarly, Gao et al. (27) reported that the risk of CLNM rises when serum TSH levels exceed 1.418 mU/L.

Based on these clinical factors, this study constructed clinical model with sex, age, and TSH level as core variables. The model achieved an AUC of 0.810 (95% CI: 0.729-0.891) in the training set and 0.792 (95% CI: 0.67-0.913) in the validation set. These results are consistent with those reported by Liu et al (26).

The radiomics model incorporated four features derived from the gray-level size-zone matrix, neighborhood gray-tone difference matrix, and gray-level co-occurrence matrix. These features describe lesion heterogeneity, regional size patterns, and texture contrast. The radiomics model achieved an AUC of 0.735 in the validation set and showed higher specificity than the clinical model. Adding Radscore to sex, age, and TSH produced the clinical-radiomics model, which achieved AUCs of 0.886 in the training set and 0.867 in the validation set, higher than either component model. Consistent with previous studies (13), incorporation of the radscore model into clinical model significantly improved predictive performance. A nomogram based on the combined model was constructed to facilitate individual risk estimation.

This study has several limitations. First, it was a single-center retrospective study with a small sample, and 170 lesion-level observations were derived from 158 patients. Multiple lesions from the same patient were treated as independent, without cluster-robust estimation, which may underestimate uncertainty. Second, performance was assessed with a single random internal split; repeated or nested cross-validation and external validation were not performed. Third, images were acquired using multiple ultrasound systems, but scanner-specific stratification and ComBat harmonization were not available, so scanner-related feature variability may have affected model stability. Fourth, only single two-dimensional images were analyzed, and multimodal ultrasound techniques were not included. Finally, TSH was modeled linearly without a formal nonlinear assessment. Larger multicenter prospective studies with clustering-adjusted analyses, scanner harmonization, and external validation are needed before clinical application.

## 5 Conclusion

Integrating ultrasound radiomics with sex, age, and TSH improved preoperative prediction of CLNM in this cohort. This individualized, non-invasive nomogram may complement conventional ultrasound assessment and support more informed preoperative decision-making in patients with PTC, although multicenter patient-level validation remains necessary.

## Data Availability

The raw data supporting the conclusions of this article will be made available by the authors, without undue reservation.

## References

[1] Agyekum EA Ren YZ Wang X Cranston SS Wang YG Wang J . Evaluation of cervical lymph node metastasis in papillary thyroid carcinoma using a clinical-ultrasound radiomic machine-learning model. Cancers (Basel). (2022) 14 (21):5266. doi: 10.3390/cancers14215266 PMC9655605 36358685

[2] Yao S Shen P Dai F Deng L Qiu X Zhao Y . Thyroid Cancer Central Lymph Node Metastasis Risk Stratification Based on Homogeneous Positioning Deep Learning. Research (Wash D C). (2024) 7:0432. doi: 10.34133/research.0432 PMC11334714 39165637

[3] Fu J Liu J Wang Z Qian L . Predictive Values of Clinical Features and Multimodal Ultrasound for Central Lymph Node Metastases in Papillary Thyroid Carcinoma. Diagnostics. (2024) 14 (16):1770. doi: 10.3390/diagnostics14161770 PMC11353660 39202260

[4] Zhang XY Zhang D Zhou W Wang ZY Zhang CX Li J . Predicting lymph node metastasis in papillary thyroid carcinoma: radiomics using two types of ultrasound elastography. Cancer Imaging. (2025) 25 (1):13. doi: 10.1186/s40644-025-00832-w PMC11827213 39948651

[5] Xiao X Chen X Li J Li P Zhu Y . Microwave ablation for lymph node metastasis in thyroid cancer: the impact of lymph node diameter. Front Endocrinol (Lausanne). (2024) 15:1430693. doi: 10.3389/fendo.2024.1430693 39165510 PMC11333885

[6] Xiao J Jiang J Chen W Hong T Li B He X . Combination of ultrasound and serological tests for detecting occult lateral lymph node metastases in medullary thyroid cancer. Cancer Med. (2023) 12 (10):11417–11426. doi: 10.1002/cam4.5856 PMC10242362 37004158

[7] Chotigavanich C Ongard S Metheetrairut C Wongsuwan P Sureepong P . Central Neck Lymph Node Size Measured by Ultrasound Significantly Predicts Central Neck Lymph Node Metastasis of Papillary Thyroid Carcinoma. Ear Nose Throat J. (2026) 105 (7):NP558–NP565. doi: 10.1177/01455613231215039 38099484

[8] Wang D Hu J Deng C Yang Z Zhu J Su X . Predictive nomogram for central lymph node metastasis in papillary thyroid microcarcinoma based on pathological and ultrasound features. Front Endocrinol (Lausanne). (2023) 14:1108125. doi: 10.3389/fendo.2023.1108125 37484943 PMC10358981

[9] Baud G Chereau N De Ponthaud C Santucci N Sebag F Goudet P . Scope of prophylactic lymph node dissection in the surgical treatment of differentiated thyroid cancer. Recommendations of the AFCE (Association francophone de chirurgie endocrinienne) with the SFE (Societe francaise d'endocrinologie) and the SFMN (Societe francaise de medecine nucleaire). J Visc Surg. (2023) 160:S84–7. doi: 10.1016/j.jviscsurg.2023.04.003 37127470

[10] Fang F Gong Y Liao L Ye F Zuo Z Li X . Value of Contrast-Enhanced Ultrasound for Evaluation of Cervical Lymph Node Metastasis in Papillary Thyroid Carcinoma. Front Endocrinol (Lausanne). (2022) 13:812475. doi: 10.3389/fendo.2022.812475 PMC8850786 35185795

[11] Hou ZK Zhao J Zhang M Hou W Li Y Yang Y . Preoperative Identification of Papillary Thyroid Carcinoma Subtypes and Lymph Node Metastasis via Deep Learning-Assisted Surface-Enhanced Raman Spectroscopy. ACS Nano. (2025) 19 (23):21807–21819. doi: 10.1021/acsnano.5c05698 40464771

[12] Zhao S Yue W Wang H Yao J Peng C Liu X . Combined Conventional Ultrasound and Contrast-Enhanced Computed Tomography for Cervical Lymph Node Metastasis Prediction in Papillary Thyroid Carcinoma. J Ultrasound Med. (2023) 42 (2):385–398. doi: 10.1002/jum.16024 35634760

[13] Shen P Yang Z Sun J Wang Y Qiu C Wang Y . Explainable multimodal deep learning for predicting thyroid cancer lateral lymph node metastasis using ultrasound imaging. Nat Commun. (2025) 16 (1):7052. doi: 10.1038/s41467-025-62042-z PMC12316972 40750786

[14] Wang G Nie F Wang Y Wang P Wang L Fan X . Value of Echogenic Foci in Diagnosing Papillary Thyroid Carcinoma and Predicting Aggressive Biological Behavior. J Ultrasound Med. (2022) 41 (5):1237–1245. doi: 10.1002/jum.15815 34415647

[15] Holoubek SA Sippel RS . Lymph node imaging for thyroid cancer. Clin Endocrinol (Oxf). (2024) 100:96–101. doi: 10.1111/cen.14993 38009335

[16] Wu X Li M Cui XW Xu G . Deep multimodal learning for lymph node metastasis prediction of primary thyroid cancer. Phys Med Biol. (2022) 67 (3):035008. doi: 10.1088/1361-6560/ac4c47 35042207

[17] Xue J Qu N Liu H Bi M Cao X . Value of multimodal ultrasound combined with BRAF gene in evaluating cervical lymph node metastasis of papillary thyroid microcarcinoma. Ultrasound Med Biol. (2024) 50:1183–7. doi: 10.1016/j.ultrasmedbio.2024.04.005 38704301

[18] Chen J Li XL Zhao CK Wang D Wang Q Li MX . Conventional Ultrasound, Immunohistochemical Factors and BRAF(V600E) Mutation in Predicting Central Cervical Lymph Node Metastasis of Papillary Thyroid Carcinoma. Ultrasound Med Biol. (2018) 44 (11):2296–2306. doi: 10.1016/j.ultrasmedbio.2018.06.020 30100099

[19] Ogle S Merz A Parina R Alsayed M Milas M . Ultrasound and the evaluation of pediatric thyroid malignancy: current recommendations for diagnosis and follow-up. J Ultrasound Med. (2018) 37:2311–24. doi: 10.1002/jum.14593 29575028

[20] Issa PP Mueller L Hussein M Albuck A Shama M Toraih E . Radiologist versus Non-Radiologist Detection of Lymph Node Metastasis in Papillary Thyroid Carcinoma by Ultrasound: A Meta-Analysis. Biomedicines. (2022) 10 (10):2575. doi: 10.3390/biomedicines10102575 PMC9599420 36289838

[21] Chen H Zhu L Zhuang Y Ye X Chen F Zeng J . Prediction model of cervical lymph node metastasis in papillary thyroid carcinoma. Cancer Control. (2024) 31:10732748241295347. doi: 10.1177/10732748241295347 39425895 PMC11497514

[22] Jia W Cai Y Wang S Wang J . Predictive value of an ultrasound-based radiomics model for central lymph node metastasis of papillary thyroid carcinoma. Int J Med Sci. (2024) 21:1701–9. doi: 10.7150/ijms.95022 39006837 PMC11241091

[23] Yan X Mou X Yang Y Ren J Zhou X Huang Y . Predicting central lymph node metastasis in patients with papillary thyroid carcinoma based on ultrasound radiomic and morphological features analysis. BMC Med Imaging. (2023) 23 (1):111. doi: 10.1186/s12880-023-01085-4 PMC10463837 37620767

[24] Zhao L Sun X Luo Y Wang F Lyu Z . Clinical and pathologic predictors of lymph node metastasis in papillary thyroid microcarcinomas. Ann Diagn Pathol. (2020) 49:151647. doi: 10.1016/j.anndiagpath.2020.151647 33126150

[25] Liu J Yu J Wei Y Li W Lu J Chen Y . Ultrasound radiomics signature for predicting central lymph node metastasis in clinically node-negative papillary thyroid microcarcinoma. Thyroid Res. (2024) 17 (1):4. doi: 10.1186/s13044-024-00191-x PMC10875890 38369523

[26] Liu W Zhang D Jiang H Peng J Xu F Shu H . Prediction model of cervical lymph node metastasis based on clinicopathological characteristics of papillary thyroid carcinoma: a dual-center retrospective study. Front Endocrinol (Lausanne). (2023) 14:1233929. doi: 10.3389/fendo.2023.1233929 PMC10519787 37766691

[27] Gao Y Qu N Zhang L Chen JY Ji QH . Preoperative ultrasonography and serum thyroid-stimulating hormone for predicting central lymph node metastasis in thyroid nodules diagnosed as or suspicious for papillary thyroid microcarcinoma. Tumour Biol. (2016) 37:7453–9. doi: 10.1007/s13277-015-4535-3 26678888

