# Supplementary material for: The clinical value of ultrasound radiomics combined with clinical data nomogram in predicting cervical lymph node metastasis of papillary thyroid carcinoma
Source: Front Oncol. 2026 Oct 7;16:1811434. doi: 10.3389/fonc.2026.1811434 (PMC13683127; doi:10.3389/fonc.2026.1811434)
Supplement: Supplementary file 1 [file DataSheet1.docx]

**Supplementary Figure S1.** Representative clinical case and individualized prediction using the clinical-radiomics nomogram. The patient was a 25-year-old man with a 10-mm thyroid lesion, a serum TSH level of 2.393 mU/L, and a Radscore of 0.534. The vertical lines indicate the points assigned to sex, age, TSH, and Radscore. The points were summed and mapped to an estimated CLNM probability of 95.6%. Histopathological examination confirmed the presence of CLNM.
